# Retraction: Structure-based virtual screening of *Trachyspermum ammi* metabolites targeting acetylcholinesterase for Alzheimer’s disease treatment

**DOI:** 10.1371/journal.pone.0324094

**Published:** 2025-05-08

**Authors:** The PLOS One Editors

The *PLOS One* Editors retract this article [1] due to concerns about peer review integrity, authorship, and potential manipulation of the publication process. These concerns call into question the validity of the reported results. We regret that the issues were not identified prior to the article’s publication.

SG agreed with the retraction. MSM responded but expressed neither agreement nor disagreement with the editorial decision. MTI, WB, MSH, MSSR, IB, and YAM either did not respond directly or could not be reached.
